# Postnatal mothers’ knowledge, perceptions and sociocultural influences on donor human milk acceptance in Richards Bay, KwaZulu-Natal, South Africa

**DOI:** 10.3389/fpubh.2026.1984123

**Published:** 2026-10-20

**Authors:** Leonora Luleka Mhlauli, Laston Gonah, Mirabel Kah-Keh Nanjoh, Sibusiso Cyprian Nomatshila, Ziphelele Peter

**Affiliations:** 1 School of Public Health, Faculty of Health Sciences, Walter Sisulu University, Mthatha, South Africa; 2 Society and Health Research Institute, Faculty of Health Sciences, Walter Sisulu University, Mthatha, South Africa

**Keywords:** breastfeeding, donor human milk (DHM), human milk banking, maternal perceptions, milk donation, postnatal mothers, qualitative research, South Africa

## Abstract

**Background:**

Donor human milk (DHM) is a critical alternative for vulnerable infants when maternal milk is unavailable, but its clinical utilisation in South Africa is constrained by limited understanding of how maternal knowledge, perceived safety, cultural meanings and social contexts shape local acceptance.

**Aim:**

To explore postnatal mothers’ knowledge, perceptions and sociocultural influences shaping acceptance of DHM in Richards Bay, KwaZulu-Natal.

**Methods:**

A qualitative design informed by interpretive phenomenology was used. Twenty-two postnatal mothers were purposively recruited from a private hospital. Semi-structured interviews were conducted in English or isiZulu, and data were transcribed, translated where necessary, and analysed using reflexive thematic analysis supported by reflexivity, peer debriefing, member checking and an audit trail.

**Results:**

Four themes were developed. DHM was largely unfamiliar, with participants reporting limited prior information and little exposure through routine maternal healthcare. Safety concerns centred on infection, donor health, screening, storage and milk processing, although confidence in healthcare institutions facilitated acceptance for some mothers. Acceptance was also shaped by maternal identity, emotional attachment to breastfeeding, spiritual and cultural beliefs, and family and community influence. Limited awareness did not equate to fixed rejection: after DHM was explained, participants distinguished between receiving and donating: willingness to receive DHM for their own infants was conditional on safety assurance and provider recommendation, whereas willingness to donate was expressed more readily and was often motivated by altruism and reciprocity. These findings indicate an interaction between informational uncertainty, institutional trust, cultural meaning and social legitimacy in shaping DHM decisions.

**Conclusion:**

Supporting informed decision-making about DHM, and potentially its uptake, requires more than increasing awareness. Routine antenatal and postnatal education, provider training, transparent communication about safety, and culturally responsive family and community engagement may help convert conditional openness into informed acceptance and donation.

## 1 Introduction

Breastfeeding provides optimal nutrition and immunological protection in early life, and the World Health Organization (WHO) recommends exclusive breastfeeding for the first 6 months of life (1, 2). For low- and very-low-birth-weight infants whose mothers’ own milk is unavailable or insufficient, the WHO recommends donor human milk (DHM) in preference to infant formula where safe milk banking facilities are available or can be established (3). Compared with formula, DHM is associated with better feeding tolerance and lower rates of necrotising enterocolitis, bronchopulmonary dysplasia and late-onset sepsis, and has been linked to improved neurodevelopmental and metabolic outcomes (4, 5). Despite this evidence, DHM use remains limited. Many South African mothers first learn about DHM and human milk banks (HMBs) only after admission to hospital (6), and a study in Empangeni, approximately 16 km from Richards Bay, found that mothers, healthcare workers and community members required more information before they would consider donating or accepting donor milk (7).

Exclusive breastfeeding rates in South Africa remain low: 32% of infants under 6 months of age were exclusively breastfed in the 2016 South Africa Demographic and Health Survey (8), and approximately 22% in the 2023 National Food and Nutrition Security Survey (9). The South African Breastmilk Reserve (SABR) operates approximately 70 milk banks in public and private hospitals and has supplied donor milk to more than 50,000 preterm infants; however, stigma, low public awareness and limited legal protection for breastfeeding mothers continue to constrain the service (10, 11). Suboptimal breastfeeding also carries substantial economic and population-level health costs (12). Infant-feeding decisions are, in addition, strongly shaped by sociocultural context: across sub-Saharan Africa and elsewhere, reported barriers to DHM acceptance include religious and cultural norms, fear that HIV may be transmitted through breast milk even after pasteurisation, formula marketing, and a lack of support from partners and family (13–16). In South Africa, beliefs about breastfeeding in public, notions of bodily purity and household gender dynamics add further complexity (17).

Most existing evidence on DHM acceptability in Africa is quantitative, reporting the proportions of mothers who are aware of DHM or willing to receive or donate it (15, 18–20). These surveys consistently report low knowledge alongside variable, often moderate, willingness, but they cannot explain how mothers arrive at their positions, why willingness to donate often differs from willingness to receive, or how cultural meanings, trust and family influence interact in these decisions. The few South African qualitative studies were conducted in Durban and in Limpopo Province (6, 16), and the nearby Empangeni study examined knowledge and attitudes across mothers, healthcare workers and community members (7). No study has explored how postnatal mothers in Richards Bay understand DHM, and these questions have not been examined among mothers using private maternity services. This gap matters locally: KwaZulu-Natal carries one of the highest HIV burdens in the country (21), which may heighten concerns about the safety of donor milk, and the study hospital’s neonatal intensive care unit cares for preterm and low-birth-weight infants, the group for whom DHM is recommended (3). Understanding how mothers interpret DHM is therefore a prerequisite for designing education and counselling that respond to their actual concerns. This study explored the knowledge, perceptions and sociocultural influences shaping DHM acceptance among postnatal mothers at a private hospital in Richards Bay, distinguishing willingness to receive DHM for one’s own infant from willingness to donate, with a view to informing health education, counselling and policy in this and comparable South African settings.

## 2 Materials and methods

### 2.1 Study design

This study used a qualitative design informed by interpretive (hermeneutic) phenomenology. Phenomenology is concerned with how people experience and make sense of a phenomenon in everyday life, rather than with testing hypotheses (22, 23). In the interpretive tradition associated with Heidegger and van Manen, researchers’ prior understandings are not assumed to be fully set aside (bracketed); instead, they are acknowledged and examined reflexively as part of the process through which meaning is interpreted (23, 24). The phenomenon of interest was mothers’ experience of encountering and making sense of DHM as an infant-feeding option in the early postnatal period, when feeding decisions are being made. Because most mothers had no direct experience of receiving or donating DHM, the study focused on their understandings, meanings and anticipated decisions rather than on accounts of DHM use.

Reflexive thematic analysis (RTA) was chosen as the analytic method because it is theoretically flexible and can be used within an experiential, interpretive framework (25, 26). RTA treats the researcher’s subjectivity as a resource for developing themes rather than as a bias to be eliminated, which is consistent with the interpretive phenomenological stance described above. The phenomenological orientation shaped the research question, the open, experience-focused interview guide and the analytic focus on mothers’ own meanings, while RTA provided a systematic and transparent procedure for developing patterns of shared meaning across participants. Individual semi-structured interviews were used so that the interviewer could cover planned topics while following up on issues raised by mothers themselves (27).

### 2.2 Study setting

The study took place at a private hospital in the central business district of Richards Bay, KwaZuluNatal. The hospital has served the area for more than 30 years and is a major provider of maternal and neonatal care. Its maternity service has an 18-bed ward with two delivery beds and a dedicated obstetric theatre, along with a 10-bed neonatal intensive care unit (NICU). It records about 55 deliveries a month and is staffed by four independent obstetricians, two neonatologists and one paediatrician, each seeing roughly 200 patients monthly. Richards Bay is a coastal industrial city on a 30 km lagoon of the Mhlathuze River in the uThungulu district, with an estimated population of 57,387 in 2022 and a largely working-class, predominantly isiZulu-speaking community. The site was chosen for its established obstetric and neonatal services, its potential to host a future DHM programme, and its accessibility to the study population.

### 2.3 Study population

Participants were postnatal mothers within 6 weeks of giving birth who were attending postnatal care at the study site. This period was chosen because it is when infant feeding decisions are made and settled. Mothers were eligible if they lived in or near Richards Bay, were within 6 weeks postpartum, could communicate in English or isiZulu, and gave written informed consent. Mothers were excluded if they could not communicate in either language, had a cognitive impairment affecting their capacity to consent or take part meaningfully, or worked as healthcare professionals in neonatology, paediatrics or lactation consulting, since their professional knowledge would not reflect that of mothers in the wider community.

### 2.4 Sampling, recruitment and sample size

Purposive sampling was used to recruit information-rich participants who could describe their understanding of DHM from the position of recently delivered mothers (22). Within the eligibility criteria, the sample was deliberately varied by age, parity, employment status and infant-feeding experience, so that the analysis could capture a range of perspectives rather than those of a single subgroup. Ward nursing staff identified potentially eligible mothers and gave them a brief verbal introduction to the study; mothers who were interested were referred to the principal investigator, who explained the study in full, confirmed eligibility and obtained written informed consent. Nursing staff were not informed which mothers agreed to take part, and mothers were assured that declining would not affect their care.

Sample size was guided by the concept of information power rather than by data or code saturation (28). Code saturation is usually judged as the point at which successive interviews stop generating new codes (29). Braun and Clarke argue that this logic does not fit RTA, because it treats codes as fixed units that already exist in the data and treats their repetition as evidence that the dataset is complete. In RTA, codes and themes are generated through the researcher’s interpretive engagement with the data, so there is no finite set of codes to exhaust, and new meanings could always be developed (30). For this reason, the absence of new codes was not used as a stopping criterion. Information power was considered relatively high because the study aim was focused, participants closely matched that aim, and the interviews produced in-depth dialogue (28). Because analysis ran alongside data collection, the principal investigator kept a log of the codes generated in each interview, together with analytic memos, and these were reviewed with co-authors during peer debriefing. The log served as a reflexive record of how the team’s understanding of mothers’ accounts was developing, not as a count for establishing saturation. In the early interviews, mothers’ accounts opened up new areas of meaning; as data collection progressed, later interviews mainly added depth, nuance and variation to patterns of meaning already being developed. By approximately the 18th interview, the remaining interviews were not changing the shape of the developing themes or how they addressed the research question. The decision to stop recruitment at 22 participants was therefore an interpretive judgement, reached collectively by the research team, that the dataset was sufficiently rich and varied to address the study aim and to support a coherent, well-evidenced account of the themes, rather than a claim that saturation had been reached.

### 2.5 Data collection

Data were generated through face-to-face, audio-recorded semi-structured interviews conducted by the principal investigator (LLM) in a private room at the study site. The interview guide covered seven areas: background and demographics, previous experience of infant feeding, knowledge of DHM, perceptions and attitudes, willingness to receive or to donate DHM, experiences of DHM education from healthcare providers, and community and social norms around milk donation. Interviews were held in English or isiZulu according to each mother’s preference 20 in English and 2 in isiZulu) and lasted 45 to 60 min. Field notes on nonverbal cues, context and the researcher’s own reflections were kept throughout.

*Timing and content of information about DHM.* To capture mothers’ prior knowledge without influencing it, each interview began with open questions about what, if anything, the mother already knew about DHM and where she had heard of it. Only after this section had been completed did the interviewer give a brief verbal explanation of DHM using a standard prepared script, covering what DHM is, who donates, how donors are screened (including for HIV), how donor milk is pasteurised and stored, and which infants usually receive it. The interview then explored perceptions, attitudes and willingness to receive or donate DHM. Views expressed after the explanation are therefore reported as responses to this information rather than as spontaneous prior knowledge, and this distinction is made explicit in the Results.

*Interviewer positionality and minimising bias.* The principal investigator is a nurse manager who lives in Richards Bay and conducted interviews in both English and isiZulu. She was not involved in the clinical care of any participant. Her familiarity with the setting and shared language helped to build rapport, but her professional identity also carried a risk that mothers would give the answers they thought a health professional wanted to hear. To reduce social desirability and interviewer bias, she introduced herself as a postgraduate researcher rather than as a member of hospital staff, stressed at the start of each interview that there were no right or wrong answers and that participation would not affect care, used open and non-leading questions, avoided expressing approval or disapproval of responses, and explored prior knowledge before giving any information about DHM. She recorded her assumptions and reactions in a reflexive journal after each interview, and these entries were discussed during peer debriefing (31, 32).

*Transcription and translation.* All interviews were transcribed verbatim by the principal investigator and checked against the recordings. IsiZulu interviews were transcribed in isiZulu and then translated into English by the principal investigator. To check accuracy, a second bilingual (isiZulu–English) speaker with a health sciences background, who was not otherwise involved in the study, independently compared the 2 translated transcripts with the isiZulu originals, and discrepancies were resolved through discussion. Culturally embedded expressions, particularly those relating to spirituality, the body and motherhood, were translated for meaning rather than word for word; where no direct English equivalent existed, the isiZulu expression was retained in the transcript with an explanatory note so that its meaning could be considered during coding (33). Quotations from isiZulu interviews are presented in English translation.

### 2.6 Data analysis

Data were analysed using Braun and Clarke’s (25) six-phase reflexive thematic analysis, working iteratively alongside data collection. The principal investigator first read and re-read the transcripts, returning to the recordings where needed and noting first impressions in analytic memos. Initial codes were then generated across the full dataset, capturing both what mothers said directly and what was implied, with NVivo used to organise and retrieve coded segments. Codes were grouped into candidate themes, which were checked against the coded extracts and the whole dataset for coherence and distinctiveness; themes with thin support or unclear boundaries were merged, refined or dropped. The remaining themes were then named and written up, with verbatim quotations used to keep the interpretation anchored in mothers’ own words. To deepen and challenge the interpretation, a second researcher read about 30% of the transcripts alongside the coded extracts and candidate themes and discussed them with the principal investigator. These discussions were not intended to reach agreement on codes or to measure consistency between coders, which would reflect a coding-reliability logic rather than RTA; instead, alternative readings were used to question assumptions, refine theme boundaries and strengthen the interpretive account (26). Consistent with the interpretive stance described in Section 2.1, the investigator did not attempt to bracket her position as a healthcare professional and community member in Richards Bay; instead, she used reflexive journaling and team discussion to examine how this position might shape her interpretation. Willingness to receive DHM and willingness to donate were coded separately because early analysis showed that mothers reasoned about them differently.

### 2.7 Trustworthiness and reflexivity

Trustworthiness was assessed against Lincoln and Guba’s (34) four criteria. Credibility was built through prolonged engagement with the setting, member checking and peer debriefing: the principal investigator conducted every interview herself, mothers were offered the chance to check summaries of their responses, and a colleague experienced in qualitative research reviewed the developing codes and early interpretations and prompted the principal investigator to reflect on how her own assumptions might be shaping them. Transferability was supported by describing the setting, the participants and the procedures in enough detail for readers to judge whether the findings apply elsewhere, and by sampling for variation in age, parity, employment status and feeding experience. Dependability rested on an audit trail recording every methodological and analytical decision, and on piloting the interview guide with two mothers; minor wording changes followed the pilot, the pilot data were excluded from the final analysis, and the revised guide was then used consistently across all interviews. Confirmability was supported by reflexive journaling, in which the investigator recorded her assumptions and reactions after each interview, and by grounding every interpretation in verbatim quotations so that the path from raw data to conclusion remains visible. The research followed the ethical requirements of Walter Sisulu University and of the participating hospital group throughout, as set out in Section 2.8.

### 2.8 Ethical considerations

Ethical approval was granted by the Walter Sisulu University Health Research Ethics Committee (Reference Number: HREC180.25) before data collection began, and permission to conduct the study was obtained from the KwaZulu-Natal Department of Health and the management of the participating hospital. Every prospective participant received an information sheet in a language she understood, setting out the purpose, procedures, risks and benefits of the study, and had time to ask questions before deciding. Written informed consent was obtained from all participants. Participation was voluntary and mothers were free to withdraw at any point without affecting the care they received. Names were replaced with unique identifier codes, data were stored securely with access limited to the research team, and no identifying information appears in the reporting. The study was conducted in line with the Declaration of Helsinki.

## 3 Results

### 3.1 Participant characteristics

Twenty-two postnatal mothers participated in the study. Participants ranged in age from 19 to 42 years, with 12 (54.55%) aged below 30 years and 10 (45.45%) aged 30 years or older. Twelve participants (54.55%) were primiparous and 10 (45.45%) were multiparous. Most participants were single (*n* = 14, 63.64%), while seven (31.82%) were married and one (4.55%) was engaged. Educational attainment ranged from NQF Level 4 to university degree, with 12 participants (54.55%) holding a university degree. Fourteen participants (63.64%) were employed. Detailed demographic and socioeconomic characteristics are presented in Table 1. Age was reported in two categories (below 30 years and 30 years or older) because, with 22 participants at a single site, finer age bands would each have contained very few mothers and could have compromised anonymity; a cut-off of 30 years divided the sample almost evenly. The exact age of each participant is given with her quotations in the sections that follow.

**Table 1:** Demographic and socioeconomic characteristics of the study participants (*N* = 22).

Characteristics of interest	Count (n)	Proportion (%)
Age category (years)		
< 30	12	54.55
≥ 30	10	45.45
Range (minimum–maximum)	19–42	–
Parity		
Primiparous	12	54.55
Multiparous	10	45.45
Grand multiparous	0	0.00
Range (minimum–maximum)	1–4	–
Marital status		
Single	14	63.64
Engaged	1	4.55
Married	7	31.82
Highest educational level		
NQF Level 4 (Matric / Grade 12 / N3)	4	18.18
Diploma	6	27.27
University degree	12	54.55
Employment status		
Unemployed	4	18.18
Internship	2	9.09
Self-employed	2	9.09
Employed	14	63.64

### 3.2 Overview of themes

Four interrelated themes were developed from the interviews: (1) limited awareness and informational preparedness; (2) perceptions of safety, risk and trust in donor human milk; (3) cultural, emotional and relational meanings of donor human milk; and (4) social norms, community influence and willingness to donate. Although participants differed in the extent to which they expressed concern about DHM, their accounts converged around a common pattern: DHM was largely unfamiliar, uncertainty about its safety was closely linked to limited knowledge, and acceptance was negotiated through maternal identity, trust, cultural meanings and social relationships. Importantly, unfamiliarity did not consistently translate into rejection. Once DHM was explained, many mothers expressed conditional willingness to accept or donate it.

### 3.3 Theme 1: limited awareness and informational preparedness

A dominant finding across the interviews was the near absence of prior knowledge about DHM. Most mothers had never encountered the concept before the interview, while those who had some awareness generally had incomplete or informal information. This limited informational preparedness appeared to shape subsequent perceptions of safety and willingness to consider DHM.

#### 3.3.1 Unfamiliarity with donor human milk

There was marked convergence across participants regarding their lack of awareness. Most mothers reported that they had never heard of DHM or did not know that human milk could be formally donated, processed and provided to another infant. One participant described her first exposure to the concept during the interview:

“I never heard about it. It’s my first time knowing that there is something called donor human milk.” (Participant 10, 19 years, single, unemployed).

Similar unfamiliarity was expressed by other participants. Importantly, participants’ accounts suggested that their lack of knowledge reflected absence of exposure rather than an established opposition to DHM. After the standard explanation of DHM had been given during the interview (Section 2.5), several mothers reconsidered their initial reservations. Another participant, for example, stated:

“I did not know that existed... but now it actually makes sense. If I’m starting to think about it, then some people can donate milk.” (Participant 2, 26 years, single, unemployed).

The convergence suggests a generalised lack of familiarity with DHM as an infant-feeding option among the participants. The reported transition from unfamiliarity to recognition of its potential value was therefore evident within the interviews themselves.

#### 3.3.2 Informal and fragmented sources of information

Among the few mothers who had encountered information about DHM, exposure was predominantly informal and mediated through social media rather than through routine maternal or neonatal healthcare. One participant explained:

“Social media. It’s all over social media. Lots of case studies. And obviously with all the recalled formula on the market at the moment, it’s more out there... Facebook, Instagram, TikTok.” (Participant 6, 27 years, engaged, employed).

Most participants consistently referred to social media or indirect sources of information. However, these accounts differed from the more complete knowledge required for informed decision-making. Participants described information that was fragmented, externally generated and not necessarily contextualised within the South African healthcare system. Thus, where awareness existed, it did not necessarily equate to adequate knowledge of DHM safety, screening or eligibility.

#### 3.3.3 Absence of healthcare-provided education

Participants showed striking convergence in reporting that DHM had not been incorporated into routine maternal health education. None reported receiving substantive information about DHM from a nurse, midwife or doctor during pregnancy, delivery or the postnatal period. One participant had encountered the topic only incidentally through a NICU nurse. One participant contrasted the emphasis on breastfeeding with the absence of information about donor milk:

“They just talk to you about breastfeeding. That’s all they talk about, and how good it is for you. But not ever about donating or taking from someone.” (Participant 5, 37 years, single, employed).

Some participants similarly described the absence of provider-led information. Several mothers proposed that information should be introduced during pregnancy rather than only after delivery, suggesting that the timing of education was itself important. The consistency of these accounts suggests that the information deficit was not simply an individual knowledge gap but also reflected a broader gap in routine communication about DHM within maternal care.

### 3.4 Theme 2: perceptions of safety, risk and trust in donor human milk

Participants’ views about DHM safety were heterogeneous, ranging from implicit confidence in hospital screening to strong concerns about infection and milk quality. However, these apparently divergent positions shared an important underlying feature: mothers’ assessments of safety were closely related to how much they knew, or believed they knew, about the processes governing donor selection, screening, processing and storage.

#### 3.4.1 Trust in healthcare-based screening

Some participants expressed confidence that DHM would be safe because it was provided through a healthcare institution. This confidence was often implicit rather than based on detailed knowledge of milk-bank procedures. One participant explained:

“I don’t have concerns because I believe that whichever hospital, whether public or private, they would have screened the milk. So, I don’t have concerns for me.” (Participant 3, 32 years, single, employed).

Some participants expressed similar assumptions. Their accounts contrasted with those of mothers who were concerned about infection and contamination. The difference was therefore not necessarily a difference in the perceived importance of safety, but rather in the degree of trust placed in healthcare institutions to manage that safety. Trust functioned as a substitute for technical knowledge for some mothers, whereas the absence of such trust or information generated uncertainty for others.

#### 3.4.2 Fear of infection and uncertainty about screening

Fear of infection was the most consistently expressed safety concern, with HIV particularly prominent. Participants also mentioned tuberculosis and the possibility of errors during donor screening. Participant 7 explained:

“There are many infections out there. If the baby is receiving the milk from another mother, especially if unscreened, may be risky... The babies may get infection as we do not know how the milk gets screened.” (Participant 7, 42 years, single, employed).

Participants consistently expressed comparable concerns. However, the accounts indicated that fear was often associated with uncertainty about the screening process rather than an unequivocal rejection of DHM. Participants generally could not describe how donors were screened or how donated milk was processed. One participant’s tentative account illustrates this uncertainty:

“I assume that they check for infection and HIV. The milk is pumped out. Maybe heated.” (Participant 1, 22 years, single, employed).

Most participants similarly provided tentative descriptions. The divergence between mothers who assumed that screening occurred and those who feared that milk might be unsafe therefore appeared to arise from different levels of confidence in a process that few could actually explain.

#### 3.4.3 Concerns about donor health, milk quality and processing

Safety concerns extended beyond infectious disease. Participants also questioned donors’ health behaviours, storage conditions, expiry dates and the possibility that screening might fail to identify a health risk. Participant 10 focused specifically on donor health:

“My concerns would be whether the person that donated was a healthy person, because you cannot donate milk if you use habit forming substances, like drinking alcohol, all of those things. So, my concern would be the health status of a donor.” (Participant 10, 19 years, single, unemployed).

Several participants raised related concerns about milk storage, expiry dates, and the thoroughness of donor screening and testing. Thus, although mothers differed in the specific risks they identified, their concerns converged around a broader question of whether the donor and the milk had been adequately assessed and controlled for potential harm. At the same time, the accounts suggest that these concerns may be amenable to clear explanations of donor screening, pasteurisation, storage and quality-control procedures.

### 3.5 Theme 3: cultural, emotional and relational meanings of donor human milk

Beyond biomedical considerations, participants interpreted DHM through existing understandings of motherhood, breastfeeding, spirituality and interpersonal trust. These meanings sometimes supported acceptance and at other times created resistance. The divergence was particularly evident in how mothers understood the relationship between breastfeeding and maternal identity.

#### 3.5.1 Breastfeeding as maternal identity and emotional attachment

For several participants, breastfeeding represented more than infant nutrition. The concept was framed not merely as a medical intervention, but as an extension of motherhood and the biological relationship between mother and child. This was explicitly demonstrated by Participant 8, who described the social and emotional expectations surrounding breastfeeding:

“When you are a mother or when you give birth, you’re all going to breastfeed. If you can’t, it’s like... you’re doing something wrong, when you’re unable to do it.” (Participant 8, 26 years, single, employed).

Some participants expressed similar understandings, describing breastfeeding as an essentially maternal act. This perspective was emphasised by participants who viewed their own breast milk as uniquely connected to their baby. Another participant stated:

“I won’t give my baby anyone else’s milk, except for mine. It’s my baby, so I’m not going to give him anyone else’s milk.” (Participant 17, 35 years, married, employed).

Several participants similarly described a strong attachment to biological breastfeeding. However, this position was not universal. Some participants, while expressing attachment to their own breast milk, supported the use of DHM for mothers who could not breastfeed. This divergence is important: emotional attachment to biological breastfeeding did not necessarily translate into rejection of DHM for all circumstances. Rather, acceptance appeared to depend partly on how donor milk was positioned in relation to the mother’s own ability and responsibility to breastfeed.

#### 3.5.2 Religious and spiritual meanings of donor milk

Some participants introduced spiritual and religious interpretations that were distinct from biomedical concerns. One participant expressed concern that characteristics associated with the donor could somehow be transmitted through breast milk:

“Each person carries a different spirit. I believe they may be passed through the baby’s milk. The babies are very sensitive and babies must be protected from the outside and forces.” (Participant 1, 22 years, single, employed).

Several participants also anticipated possible religious or cultural objections, with some drawing a comparison with beliefs surrounding blood transfusion. These accounts demonstrate that decisions about DHM cannot be understood solely through biomedical concepts of safety. For some mothers, the meaning of donor milk extended to questions of spiritual identity, purity and what may be transmitted between donor and recipient. Such beliefs represented a distinct basis for hesitation from the infectionrelated concerns described in Theme 2.

#### 3.5.3 Trust in healthcare providers as a facilitator of acceptance

In contrast to concerns rooted in uncertainty or cultural hesitation, several mothers described healthcare professionals as trusted authorities whose recommendations could facilitate acceptance. One participant stated:

“I trust a health care provider. That is the profession that made me believe so much in breast milk in general. So if they give me a go ahead on that, I would probably take it. I don’t think the professionals will tell us lies or to harm our babies.” (Participant 4, 35 years, married, employed).

Several participants expressed similar views. This finding contrasts with the earlier observation that mothers had received little or no formal information about DHM. Thus, healthcare providers occupied a paradoxical position: they were highly trusted as sources of reassurance, yet DHM was largely absent from their communication with mothers. The accounts suggest that provider trust represents an important potential pathway through which information could be translated into confidence and informed decision-making.

### 3.6 Theme 4: social norms, community influence and willingness to donate

Participants’ decisions were also situated within family, peer and community relationships. Social influence could reinforce uncertainty and stigma, but it could also support acceptance and donation. The same social networks that mothers identified as potential sources of resistance could therefore also function as sources of encouragement.

#### 3.6.1 Family and peer influence on feeding decisions

Family and friends were described as influential in shaping maternal decisions about infant feeding. One participant acknowledged that negative opinions from close social contacts could change her own position:

“I am someone who’s easily convinced by family and friends. So if they are very negative towards it, then I’ll start having doubts. So I would probably believe what they’re saying at that moment.” (Participant 4, 35 years, married, employed).

Other participants similarly described social influence, but some also identified positive examples within their social networks. Some participants viewed milk donation through the broader principle of communal responsibility for children, while others described a friend’s sister who had provided breast milk to a relative’s newborn. These contrasting accounts indicate that social networks did not exert a uniformly negative influence. Rather, familiarity with supportive forms of milk sharing appeared capable of normalising the idea of providing human milk to another infant.

#### 3.6.2 Perceived social acceptability and representation of milk donation

Participants also described broader social perceptions that could discourage DHM use or donation. Some linked difficulties with breastfeeding to perceptions of maternal failure, while others viewed milk donation as something associated with groups outside their own communities. One participant stated:

“It’s people that donate, normally white people. I’ve never seen a black person donate... With all the videos I’ve seen, it’s only whites.” (Participant 5, 37 years, single, employed).

Several participants similarly described stigma surrounding inability to breastfeed and rejection of milk sharing. These accounts suggest that social acceptability was influenced not only by whether DHM was considered safe, but also by whether mothers perceived donation as a familiar and socially legitimate practice within their own communities. The perceived absence of local representation may therefore have reinforced the sense that DHM was unfamiliar or culturally distant.

#### 3.6.3 Altruism and willingness to donate

Despite the substantial uncertainty surrounding DHM, willingness to donate emerged as a strong and comparatively positive finding. Once the concept had been explained, several participants expressed a desire to help infants and mothers who might need donor milk. One participant explained:

“Now that I know, if I had an excess supply, I would actually donate milk. I think it’s a good thing to donate for the community. Because if I was to also be in trouble and need something, I would want someone to donate to me.” (Participant 2, 26 years, single, unemployed).

Several participants expressed similar willingness, with some framing donation in terms of saving another baby’s life. The convergence of these accounts contrasts with the strong reservations expressed elsewhere in the interviews. Mothers who initially lacked any awareness of DHM, or who expressed concerns about accepting milk for their own babies, could nevertheless describe donation as desirable once the concept was understood. This suggests that reluctance to engage with DHM was not necessarily equivalent to opposition to the underlying principle of human milk donation.

#### 3.6.4 Distinguishing willingness to receive from willingness to donate

Mothers’ accounts showed that receiving DHM for their own infant and donating their own milk were distinct decisions, grounded in different considerations. Willingness to receive was typically conditional and negotiated. It depended on assurance that the donor had been screened and the milk safely processed (Section 3.4), on a recommendation from a trusted healthcare provider (Section 3.5.3), and on whether DHM was needed because the mother could not provide enough of her own milk. For some mothers, receiving another woman’s milk conflicted with their sense of breastfeeding as an exclusively maternal act or raised spiritual concerns (Sections 3.5.1 and 3.5.2), and some stated that they would give their baby only their own milk. Willingness to donate, by contrast, was expressed more readily once DHM had been explained. It was framed in terms of altruism, reciprocity and helping another baby, and was usually conditional on the mother having milk to spare. Accounts of donation rarely referred to the safety concerns that dominated discussion of receiving, perhaps because donating did not require mothers to accept risk on behalf of their own infant. As a result, some mothers who had reservations about giving donor milk to their own babies nevertheless described themselves as willing to donate.

### 3.7 Cross-theme synthesis: from unfamiliarity to conditional acceptance

Across the four themes, participants’ accounts revealed a consistent but non-linear pattern in which knowledge, perceived safety, cultural meaning, interpersonal trust and social norms interacted to shape views of DHM. The most pronounced point of convergence was the limited prior exposure to DHM in this South African study setting. This informational gap was closely connected with uncertainty about safety and screening, although participants differed in how they responded to that uncertainty. Some placed considerable trust in hospitals and healthcare professionals, whereas others required greater explanation before feeling comfortable.

Acceptance was similarly heterogeneous. For some mothers, the biological and emotional meaning of breastfeeding created a strong preference for their own milk, while others regarded DHM as an appropriate alternative when maternal milk was unavailable. Religious and spiritual interpretations provided another distinct basis for hesitation in this setting. At the social level, family and peer influence could reinforce scepticism, but positive experiences of milk sharing and perceptions of communal responsibility could facilitate acceptance.

A particularly important point of convergence emerged when DHM was explained. Several mothers who had initially been unfamiliar with or uncertain about donor milk expressed willingness to consider accepting or donating it. Donation was often framed in altruistic and reciprocal terms, suggesting that mothers could distinguish between concerns about the process and safety of DHM and the broader principle of helping another infant. The findings therefore suggest that the principal barrier was not uniformly negative attitudes toward DHM, but rather a combination of limited awareness, uncertainty about safety, culturally situated meanings and the absence of trusted, structured information. Conversely, healthcare-provider trust, clear information, supportive social networks and altruistic values appeared to provide potential pathways towards greater acceptance and willingness to donate.

Figure 1 summarises how these factors interacted. Awareness and information, perceived safety and trust in healthcare providers shaped whether mothers felt able to evaluate DHM, while maternal identity, cultural and spiritual beliefs, and family and community influence shaped whether DHM felt personally and socially acceptable. These factors bore most heavily on willingness to receive DHM for one’s own infant, whereas willingness to donate was driven mainly by altruism and reciprocity once DHM had been explained. The figure is a conceptual summary of the interpretations developed in this study and is not intended to represent a tested causal model.

**Figure 1:**
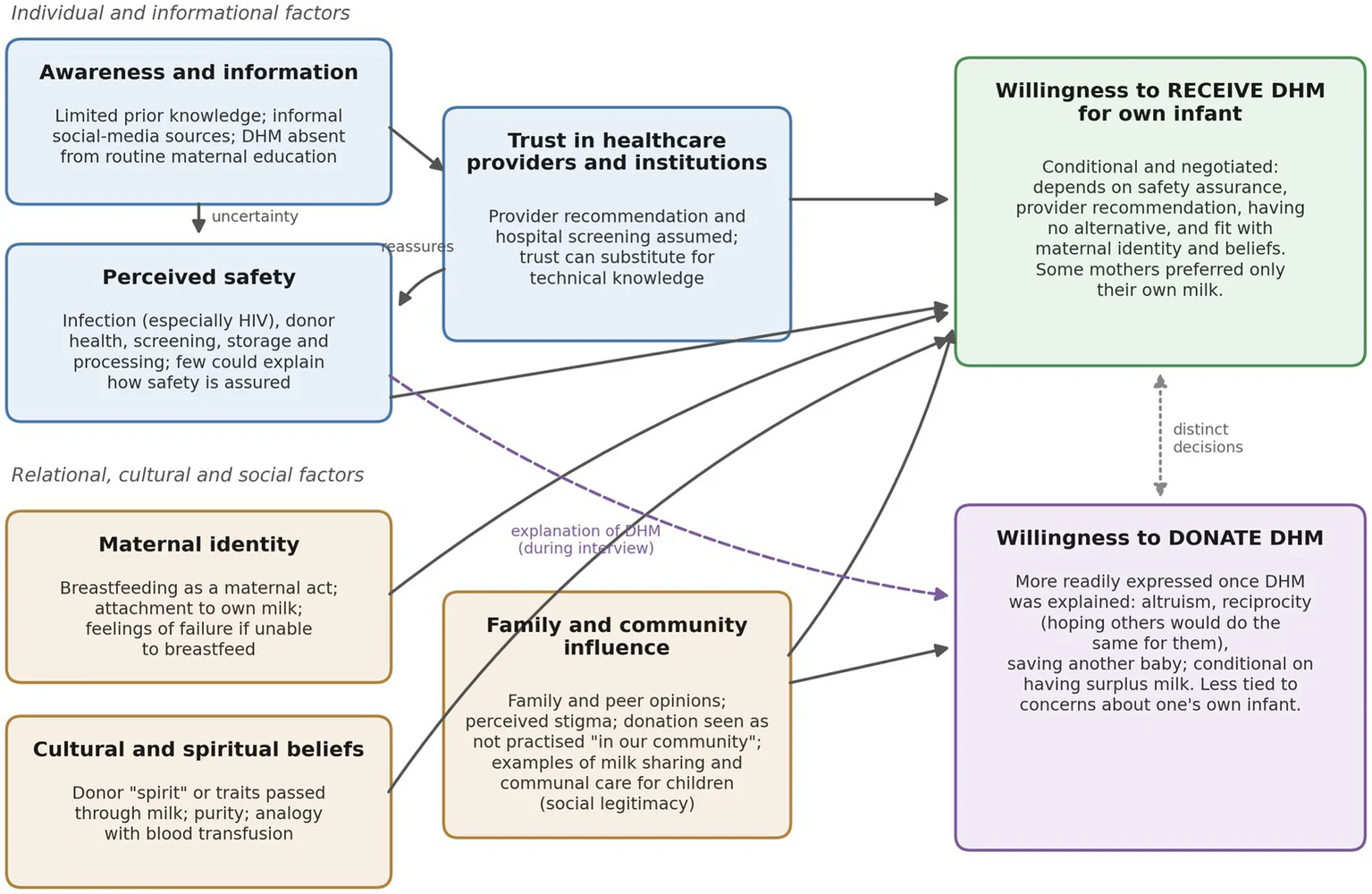
Conceptual summary of the factors shaping postnatal mothers’ willingness to receive and to donate donor human milk (DHM) in this study. Solid arrows indicate influences described by participants; the dashed arrow indicates the effect of the explanation of DHM given during the interview; the dotted arrow indicates that receiving and donating were distinct decisions.

## 4 Discussion

This study explored postnatal mothers’ knowledge, perceptions and sociocultural influences shaping acceptance of DHM within a coastal South African setting. The findings reveal a striking combination of limited prior awareness and substantial conditional openness to DHM. Most mothers had reportedly never encountered DHM before the interview, yet, once the concept was explained, several became willing to consider receiving it under certain conditions, and willingness to donate was expressed more readily. Their reservations centred primarily on safety, particularly infection, donor health, screening, storage and processing, while acceptance was also shaped by maternal identity, cultural and spiritual meanings, trust in healthcare providers, and social influence. These findings suggest that low awareness should not be equated with entrenched opposition. Rather, acceptance appeared to have been contingent on whether mothers had access to information and trusted social and professional contexts through which DHM can be understood and evaluated.

The reported absence of DHM from routine maternal health communication is therefore particularly consequential. Although participants regarded healthcare professionals and hospitals as trusted sources of information, none reported receiving substantive education about DHM during pregnancy, delivery or the postnatal period. This may create a gap between the availability of a clinical resource and its accessibility as an informed maternal choice. Existing evidence similarly indicates limited maternal awareness and gaps in provider communication regarding DHM (35, 36). This pattern is consistent with quantitative evidence from South Africa and elsewhere in Africa. In a cross-sectional survey of mothers in a high-HIV-prevalence area of KwaZulu-Natal, 64.4% had inadequate knowledge of donor breast milk (15), and in a multi-centre survey of 1,235 mothers in south-east Nigeria only 39% had heard of donor milk and 10% had adequate knowledge of it (18). In the Nigerian study, adequate knowledge was one of the few significant predictors of willingness to donate or receive donor milk (18), which is consistent with our observation that several mothers reconsidered their reservations once DHM had been explained. The present study adds to this survey evidence by showing how the knowledge gap is experienced: mothers’ information came mainly from social media rather than from healthcare providers, even though providers were regarded as the most trusted source. However, the present findings suggest that the issue extends beyond knowledge acquisition: when DHM is introduced only when an infant requires supplementation, mothers may be encountering an unfamiliar intervention at precisely the point when decisions are emotionally and clinically demanding. Introducing DHM within routine antenatal and postnatal education could instead establish familiarity before such decisions arise (37, 38). Mampane and Wolvaardt similarly recommended that mothers be educated about safe milk banking before NICU admission (6). The willingness expressed after participants received basic information further suggests that awareness may be a modifiable barrier rather than evidence of fixed resistance.

The safety concerns expressed by participants emphasise the importance of how DHM was communicated. Mothers who trusted hospital-based screening were often unable to explain the processes that made donor milk safe, while those with less confidence focused on possible infection, donor health and milk handling. These responses should not be reduced to either trust or mistrust; they reveal a trust-knowledge gap in which institutional confidence may substitute for technical understanding for some mothers, while insufficient information produces uncertainty for others. This distinction has practical significance. Technical safeguards such as donor screening, pasteurisation, storage and quality control may have little value in building maternal confidence if their purpose and operation remain invisible to mothers. Evidence identifying safety and quality concerns as barriers to DHM acceptance, particularly in settings where systems and procedures may be less familiar to the public, supports the importance of transparent communication (6, 35, 39–42). Fear of HIV transmission was likewise the most commonly stated concern in an earlier qualitative study in Durban, KwaZulu-Natal, where participants also expressed discomfort about using another mother’s milk and recommended transparency about how donor milk is sourced and prepared (16). In eastern Ethiopia, fear of disease transmission was the most frequently reported reason for unwillingness to donate or use donor milk (68.8% of 1,085 mothers), followed by concerns about unhygienic milk collection (19), and concerns about disease, including HIV, and hygiene were also reported in Nairobi (20) and eastern Uganda (36). One contrast is notable. In the Durban study, some mothers expressed mistrust of healthcare providers (16), whereas mothers in the present study generally regarded providers as trustworthy: the problem they described was not that providers were distrusted, but that providers rarely talked about DHM. This difference may partly reflect the private-sector setting of the present study, and it suggests that, here, provider counselling is an underused rather than a discredited channel. Healthcare providers therefore have a role extending beyond recommending DHM: they are a critical interface through which the safety of the milk-banking system can be understandable and credible.

However, the findings also indicate that information alone cannot resolve all uncertainty, because DHM is not understood solely as a biomedical product. For some mothers, breastfeeding was closely tied to maternal identity, biological connection and emotional attachment, while spiritual and cultural beliefs influenced how the transfer of milk between women was understood. These meanings may help explain why a mother could recognise the clinical value of DHM while remaining reluctant to give it to her own infant. Conversely, willingness to donate was often framed in terms of altruism, reciprocity and helping another child. This apparent contradiction, being willing to donate but hesitant to receive, suggests that acceptance and donation should not be treated as opposite ends of a single attitude. Donation can be understood as an altruistic act, whereas receiving another woman’s milk requires mothers to negotiate safety, trust, maternal identity and cultural meaning simultaneously (43, 44). This asymmetry is well documented in survey research. In Nairobi, 78% of participants were willing to donate milk to a human milk bank, but only 59% would allow their own children to be fed DHM (20). The pattern is not universal: in eastern Ethiopia, willingness was low for both, with 11% of mothers willing to donate and 15.2% willing to use donor milk (19), indicating that the balance between donating and receiving depends on context. The present study helps to explain why the two decisions can diverge: receiving DHM requires a mother to accept perceived risk and cultural meaning on behalf of her own infant, whereas donating allows her to act on altruistic and reciprocal values without that burden (Figure 1). DHM programmes may therefore need different messages for potential recipients, focused on safety assurance and on reconciling DHM with maternal identity, and for potential donors, focused on the practicalities of donation and its value to other infants.

Consequently, DHM programmes that rely exclusively on biomedical messaging may only partly address the decision-making process. Culturally responsive communication needs to acknowledge what breast milk represents to mothers while providing accurate information about regulated milk banking (45). Religious and cultural objections to milk sharing have also been reported in Turkey (13) and in eastern Ethiopia, where 19.1 and 14.7% of mothers cited religion and culture, respectively, as reasons for not donating or using donor milk (19). The authors of the Nigerian survey recommended involving community and religious leaders, alongside clinicians, in DHM education (18), an approach that may be particularly relevant where, as in the present study, concerns about spiritual transmission through milk coexist with biomedical concerns.

These individual perceptions and interpretations may further be shaped by the social environment in which maternal decisions occur (46). Participants described family, friends and community attitudes as capable of reinforcing either hesitation or acceptance, while familiarity with milk sharing could make donation more socially understandable. The perceived lack of local representation of milk donors is particularly relevant because it suggests that DHM may remain socially distant when mothers do not see the practice reflected within their own communities. This extends the issue beyond individual awareness to social legitimacy. A mother may be personally willing to consider DHM but still hesitate if important people around her regard it as unfamiliar or inappropriate (47, 48). Conversely, visible examples of donation and supportive community narratives may normalise the practice (49, 50). Thus, increasing DHM utilisation requires attention not only to what mothers know, but also to whether DHM has become sufficiently familiar and socially legitimate for that knowledge to translate into action.

The findings collectively point to a need to move from an information-deficit model to an informed trust model of DHM engagement (38, 46). Based on the study findings, the prevailing challenge is not simply to tell mothers that donor milk exists, but to create conditions in which they can make informed decisions about it: information needs to be introduced early, safety procedures need to be understandable, healthcare providers need to be equipped to counsel mothers, and cultural, family and community contexts need to be incorporated into communication. This is particularly relevant in South Africa, where milk-bank infrastructure exists but awareness and utilisation remain limited (10, 11, 15). The findings suggest that expanding service capacity alone may not be sufficient if mothers remain unaware of the service or uncertain about its safety and social legitimacy. Conversely, the conditional willingness evident in this study provides an important opportunity: where familiarity, trust and cultural responsiveness are deliberately built into DHM programmes, existing openness may be translated into informed acceptance and donation. The implications may therefore extend beyond the study setting to similar settings where the challenge is not only establishing safe DHM systems, but making those systems visible, trusted and meaningful to the communities they are intended to serve.

### 4.1 Study limitations

This study has several limitations. It was conducted at a single private hospital in Richards Bay with 22 purposively selected postnatal mothers, more than half of whom held a university degree and most of whom were employed. The findings are therefore contextually situated and are not statistically generalisable to mothers using public-sector services or living in other geographical and socioeconomic contexts, where access to information, trust in providers and exposure to DHM may differ.

Recruitment through ward nursing staff may have introduced selection bias. Nurses may, consciously or not, have approached mothers they considered more articulate, cooperative or interested in breastfeeding, and mothers with strong negative views about DHM, or with less positive relationships with staff, may have been less likely to be referred or to agree to take part.

Social desirability bias is also possible. Interviews were conducted by a healthcare professional within a hospital, and mothers may have expressed more favourable views about DHM, breastfeeding or healthcare providers than they held privately. In addition, the explanation of DHM provided during the interview may itself have encouraged more positive responses. The measures described in Section 2.5, together with reflexive journaling, peer debriefing, member checking, an audit trail and discussion of about 30% of the transcripts with a second researcher, were used to reduce and account for these influences but cannot eliminate them. Willingness expressed shortly after a brief explanation should therefore be interpreted as openness to DHM, not as a prediction of actual acceptance or donation.

Most participants had no direct experience of receiving or donating DHM. The findings therefore reflect mothers’ understandings, perceptions and anticipated decisions rather than lived experience of DHM use, which limits what they can reveal about how mothers experience DHM once it is offered or received.

The study captured only mothers’ perspectives. Healthcare providers, milk donors, mothers whose infants had received DHM, partners, family members and community representatives were not interviewed; accounts of provider influence, family responses and community attitudes therefore represent mothers’ perceptions of these influences. Future research should include these groups, particularly providers and actual donors and recipients.

Finally, although translation procedures were designed to preserve meaning (Section 2.5), subtle differences in meaning may have been lost when isiZulu interviews were translated into English, particularly for culturally and spiritually embedded concepts. These limitations notwithstanding, the study provides contextually grounded insight into how knowledge, trust and sociocultural meaning interact in shaping maternal consideration of DHM.

## 5 Conclusion

This study suggests that maternal acceptance of DHM is shaped by the interaction of knowledge, trust, perceived safety, maternal identity and social legitimacy, rather than by a single positive or negative attitude towards donor milk. The coexistence of limited awareness and conditional willingness to receive DHM and a more readily expressed willingness to donate suggests that unfamiliarity should not be interpreted as rejection. Instead, the findings point to a gap between DHM as a clinical resource and DHM as an accessible and socially understood option for mothers. Addressing this gap may require more than increasing awareness: DHM should be introduced through routine antenatal and postnatal education, supported by knowledgeable healthcare providers, transparent communication about safety, and culturally and socially responsive engagement. Such an approach may help translate existing conditional openness into informed acceptance and donation, while providing a stronger foundation for DHM integration in Richards Bay and comparable South African settings.

Strengthening how DHM is communicated along these lines may improve mothers’ awareness, support informed decision-making and build trust in milk banking, and could thereby increase the uptake of DHM and milk donation in Richards Bay and similar settings. Whether such improvements translate into greater DHM use and improved neonatal outcomes was beyond the scope of this study and should be evaluated in future intervention research that includes healthcare providers, donors and recipients.

## Data Availability

The raw data supporting the conclusions of this article will be made available by the authors, without undue reservation.
